# Current State of Preeclampsia Mouse Models: Approaches, Relevance, and Standardization

**DOI:** 10.3389/fphys.2021.681632

**Published:** 2021-07-02

**Authors:** Christopher A. Waker, Melissa R. Kaufman, Thomas L. Brown

**Affiliations:** Department of Neuroscience, Cell Biology, and Physiology, Boonshoft School of Medicine, Wright State University, Dayton, OH, United States

**Keywords:** placenta, preeclampsia, pregnancy, mouse, models, hypertension, proteinuria, trophoblast

## Abstract

Preeclampsia (PE) is a multisystemic, pregnancy-specific disorder and a leading cause of maternal and fetal death. PE is also associated with an increased risk for chronic morbidities later in life for mother and offspring. Abnormal placentation or placental function has been well-established as central to the genesis of PE; yet much remains to be determined about the factors involved in the development of this condition. Despite decades of investigation and many clinical trials, the only definitive treatment is parturition. To better understand the condition and identify potential targets preclinically, many approaches to simulate PE in mice have been developed and include mixed mouse strain crosses, genetic overexpression and knockout, exogenous agent administration, surgical manipulation, systemic adenoviral infection, and trophoblast-specific gene transfer. These models have been useful to investigate how biological perturbations identified in human PE are involved in the generation of PE-like symptoms and have improved the understanding of the molecular mechanisms underpinning the human condition. However, these approaches were characterized by a wide variety of physiological endpoints, which can make it difficult to compare effects across models and many of these approaches have aspects that lack physiological relevance to this human disorder and may interfere with therapeutic development. This report provides a comprehensive review of mouse models that exhibit PE-like symptoms and a proposed standardization of physiological characteristics for analysis in murine models of PE.

## Introduction

Preeclampsia (PE) is a leading cause of maternal and fetal mortality and morbidity and a major cause of preterm birth (Roberts and Lain, 1998; Sibai, 2006). Responsible for more than 76,000 maternal and 500,000 infant deaths per year worldwide, PE has short-term economic costs in the billions and even greater long-term costs when the increased risk for chronic comorbidities later in life are also considered (Martin et al., 2011; Stevens et al., 2017; Burton et al., 2019; Rana et al., 2019). Some of the well-established, long-term consequences for the mother and offspring include an increased risk of cardiovascular and metabolic disease (Bokslag et al., 2016; Alsnes et al., 2017; Paauw and Lely, 2018). Recent studies have also linked PE to potential neurocognitive deficits in offspring (Tuovinen et al., 2012; Dang et al., 2016; Rätsep et al., 2016; Mak et al., 2018). A preeclamptic pregnancy not only increases the risk of the mother developing the condition in subsequent pregnancies, but also significantly increases the risk of female offspring to develop and male offspring to contribute to the development of PE (Lie et al., 1998; Esplin et al., 2001; Dempsey et al., 2003; Innes et al., 2003; Skjaerven et al., 2005; Klebanoff, 2007; Zetterström et al., 2007; Hernández-Díaz et al., 2009; á Rogvi et al., 2012; Cheong et al., 2016; Galaviz-Hernandez et al., 2019; Maric-Bilkan et al., 2019).

Preeclampsia is thought to occur because of abnormal placental development or placental dysfunction (Roberts and Escudero, 2012; Ilekis et al., 2016; Schneider, 2017). Pathological characteristics of PE were historically defined as rapid onset pregnancy-specific hypertension with accompanying proteinuria and parturitional resolution (Hypertension in pregnancy, 2013; Rana et al., 2019). Recent reclassification now includes maternal liver damage in the absence of proteinuria (Hypertension in pregnancy, 2013). Physiological features associated with PE include alterations in placental lineage and maternal and fetal blood spaces, lack of maternal spiral artery remodeling, changes in angiogenic mediators, and elevated maternal inflammatory cytokines. Hypoxia and elevated levels of hypoxia-inducible factor 1 alpha (HIF-1α) protein have also been noted in the placenta and are strongly associated with preeclampsia (Caniggia and Winter, 2002; Rajakumar et al., 2004, 2008; Zamudio et al., 2007, 2014; Kanasaki and Kalluri, 2009; Pringle et al., 2010; Rolfo et al., 2010; Pennington et al., 2012; Korkes et al., 2017; Chang et al., 2018; Park et al., 2018; Rana et al., 2019).

The severity of the condition is variable and therapeutic intervention includes pharmacologic management of the hypertension using antihypertensives like methyldopa, labetalol, and nifedipine, as well as magnesium sulfate to prevent seizures in severe cases (Phipps et al., 2016). However, despite decades of work and numerous clinical trials, the only definitive way to resolve PE is parturition. The lack of improvement in PE treatment, in preceding years, may be due to the view of “PE” as a single condition rather than stratifying the different clinical presentations into subtypes. A stratification scheme with broad support categorizes PE as early-onset – *which typically has abnormal placental development and fetal growth restriction* and late-onset – *which generally has normal placental development and normal fetal weight* (Raymond and Peterson, 2011; Roberts and Escudero, 2012; Redman et al., 2014; Gathiram and Moodley, 2016; Staff and Redman, 2018). In addition, HELLP syndrome, characterized by hemolysis, elevated liver enzyme levels, and low platelet levels, has been identified as a rare, but severe, variant of PE (Hypertension in pregnancy, 2013; Wilkerson and Ogunbodede, 2019). However, distinct molecular subtypes remain elusive and identification of subtypes may be requisite to determining common etiologies and the development of more effective therapies.

Another factor that may have contributed to the lackluster improvement in patient outcomes may originate from the number and variety of approaches to simulate PE-like symptoms in mice. Mouse models are widely used and well-accepted in the study of PE, due to the extensive genetic characterization and high homology to humans, as well as similar hemochorial blood flow (Soncin et al., 2015; Maltepe and Fisher, 2015; Soares et al., 2018). The mouse placenta has a high degree of functional conservation and is structurally analogous to the human placenta (Cross et al., 2003; Soncin et al., 2015). Approaches that have been used to simulate PE-like symptoms in mice include surgical manipulation, mixed strain mating, genetic overexpression and knockout, exogenous agent administration, systemic adenoviral infection, and trophoblast-specific transgenesis (Table 1). These numerous approaches each produce some or all of the hallmarks of PE and some ancillary phenotypes with varying relevance to the human condition. Evaluation of the relevance and accuracy of these models is further impeded by inconsistent investigations into PE-associated pathologies, perhaps due to a lack of standardized criteria.

**TABLE 1:** Mouse models of preeclampsia.

**Model**	**Approach**	**PE subtype**	**References**
DBA/2 × CBA/J	Mixed strain	NA	Clark et al., 1998; Girardi et al., 2006; Ahmed et al., 2010
STOX1 KO	Germline mutant	Early-onset	Doridot et al., 2013; Collinot et al., 2018
IDO KO	Germline mutant	NA	Santillan et al., 2015
ELABELA KO	Germline mutant	Early-onset	Ho et al., 2017
COMT KO	Germline mutant	Early-onset	Kanasaki et al., 2008
C1q KO	Germline mutant	Early-onset	Singh et al., 2011; Agostinis et al., 2017
AT1-AA	Exogenous agent	Late-onset	Zhou et al., 2008
LPS	Exogenous agent	Early/late-onset	Li et al., 2018; Huai et al., 2018
TNF-α	Exogenous agent	Late-onset	Bobek et al., 2015; Bobek et al., 2018
TNFSF14/LIGHT	Exogenous agent	Late-onset	Wang et al., 2014; Iriyama et al., 2015
L-NAME	Exogenous agent	Early/late-onset	Yoshikawa et al., 2017
RUPP	Surgical	Late-onset	Intapad et al., 2014; Fushima et al., 2016; Natale et al., 2018; Sekimoto et al., 2020
Adenoviral sFLT-1	Maternal systemic	HELLP	Lu et al., 2007; Suzuki et al., 2009; Bergmann et al., 2010
Lentiviral sFLT-1	Trophoblast-specific	Early-onset	Kumasawa et al., 2011
Adenoviral HIF-1α	Maternal systemic	HELLP	Tal et al., 2010
Lentiviral HIF-1α	Trophoblast-specific	Early-onset	Albers et al., 2019

*Mouse models of preeclampsia without obvious pathologies prior to pregnancy or experimental intervention. Models are identified by approach and characterized as early-onset or late-onset subtypes if the models exhibited the classical hallmarks of preeclampsia in humans: hypertension and proteinuria. Determination of early onset or late-onset was based on whether experimental intervention occurred prior to or after placental development, which is finished by E13.5/14.5. The mixed early/late-onset indicates that the reported began experimental intervention just before the placenta finished forming. Not applicable (NA) – This model does not recapitulate the classical hallmarks of PE.*

**FIGURE 1:**
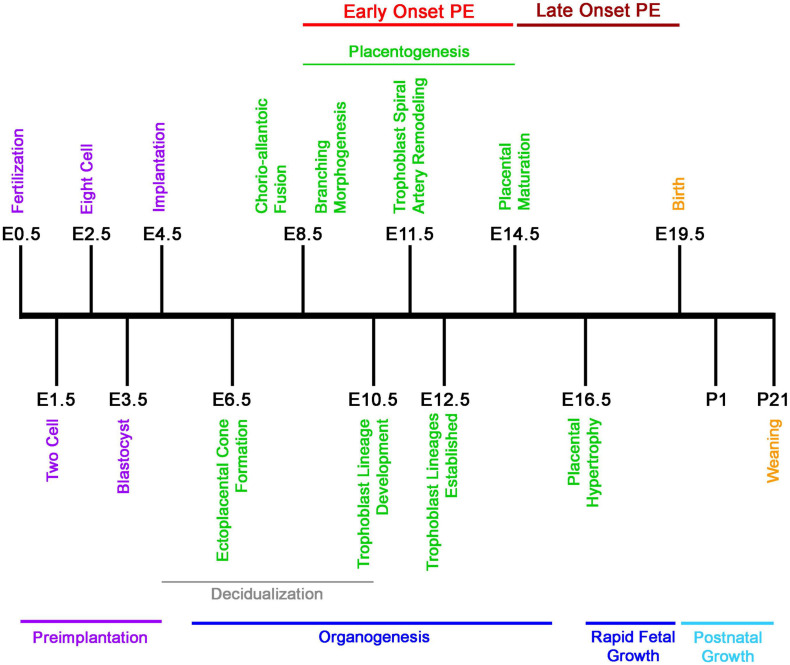
Developmental timeline of mouse pregnancy. Graphical chronology of events associated with placental and fetal development in mice (Soncin et al., 2015; Sones and Davisson, 2016). Days of development are denoted as embryonic (E) and postnatal (P).

**FIGURE 2:**
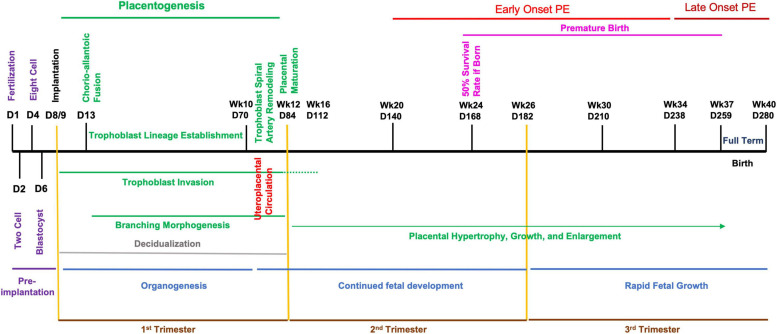
Developmental timeline of human pregnancy. Graphical chronology of events associated with placental and fetal development in humans (Benirschke et al., 2012; Soncin et al., 2015; Sones and Davisson, 2016; Blum et al., 2017). Days of development are denoted as day of development (D) and week of development (Wk).

While there are distinct differences between the mouse and human fetoplacental development, the level of functional and molecular conservation is remarkable regarding the timing of development and birth, with mouse pregnancy in the order of days versus weeks in humans (Figures 1, 2) (Benirschke et al., 2012; Soncin et al., 2015; Sones and Davisson, 2016; Blum et al., 2017). The first trimester in humans features chorioallantoic fusion, branching morphogenesis, trophoblast lineage development, spiral artery remodeling, and placental maturation which are typically finished by gestational week 12 (Wk12) and while mouse pregnancy has not been traditionally discussed in terms of trimesters, these processes occur by embryonic day 12.5 (E12.5) and have generally analogous developmental windows (Figures 1, 2). During the second trimester, fetal development and maturation continues and a similar period is ongoing in mice from E12.5 to E16.5. The third trimester in humans, starting Wk26, is characterized by rapid fetal growth and placental hypertrophy and is similar to the period beginning on E16.5 in mice until parturition (Figures 1, 2). A key difference between mouse and human generally analogous gestational timepoints is the mouse has a significantly compacted “second and third trimester” compared to the human.

In humans, the designation of early- or late-onset is determined by gestational week, with Wk34 as the delineation. PE arising before this gestational date is termed early-onset and PE occurring after as late-onset. Early-onset PE is generally thought to be associated with disruption of normal placentation. Mouse pregnancy has a well-defined developmental timeline that can used to classify PE models similar to the proposed human stratification of early-onset and late-onset depending on the gestational day of intervention and reported disruption of placental structure and lineage (Figures 1, 2) (Table 1). Determination of early-onset and late-onset was based on whether experimental intervention occurred prior to (early) or after (late) placental development, which is finished by E13.5/E14.5. However, a “late-onset” approach does not necessarily mean that placental structure and lineage are absent of pathology, and these elements should be investigated in every approach (Natale et al., 2018).

In this review, reported pre-pregnancy, normotensive approaches to simulate PE-like symptoms in mice are discussed. Models of PE with pre-existing comorbidities such as chronic hypertension, diabetes, and obesity have not been included in this review because these models require extensive and specific discussion to compare the pregnancy-specific phenotype with that of the non-pregnant condition and place these findings with the unique context of the associated human conditions. The central findings of the reported models are discussed, along with the relevance to the human condition, as well as the advantages and drawbacks. The goal of this comprehensive review is to provide researchers studying PE with an overview of the current state of mouse models, their relevance to the human condition, and to propose a standardization of physiological analysis in the development and use of PE models.

## The Approaches

### Mixed Strain Approach

#### DBA/2 × CBA/J

When a male DBA/2 inbred mouse is mated to a CBA/J inbred female, the resultant first pregnancies are highly abortion prone, with approximately one-third of the fetuses undergoing reabsorption (Bobé et al., 1986; Clark et al., 1998; Ahmed et al., 2010). The resorptions are characterized by focal necrosis at the fetal trophoblast-decidual junction with corresponding thrombosis and hemorrhage of nearby large vessels (Clark et al., 1998). These pregnancies also exhibit significant complement deposition on trophoblast giant cells, as early as embryonic day 6 (E6), with maternal renal dysfunction as early as E10 and before the placenta has completely formed.

Abnormal placental development was present with a decrease in trophoblast giant cells and a reduced presence of fetal vessels in the labyrinth. Increased soluble fms-like tyrosine kinase-1 (sFLT-1) and reduced vascular endothelial growth factor (VEGF) were observed in maternal plasma, suggestive of angiogenic dysregulation. Elevation of plasma tumor necrosis factor alpha (TNF-α) levels and monocyte invasion of the decidua were also observed. Surviving offspring exhibited fetal growth restriction and were approximately 30% smaller than controls (Girardi et al., 2006). However, maternal blood pressure was not elevated, which is uncharacteristic of PE, although proteinuria was observed (Ahmed et al., 2010). Treatment of pregnant DBA/2 × CBA/J females with complement C3 or C5 inhibitors prevented the phenotypic pathology and fetal growth restriction, suggesting that the complement pathway is a major mediator of this phenotype. DBA/2 mice have an H-2K(d) MHC class 1 antigen, while CBA/J mice have a H-2K(k) MHC class 1 antigen; thus, crossing these strains likely leads to immune rejection of the semiallogeneic placenta and cytokine-mediated abortion resulting from partial MHC incompatibility. The immune rejection of the placenta and fetus, as well as the lack of hypertension in DBA/2 × CBA/J pregnancies, does not resemble PE in humans, but may more closely represent a model of MHC incompatibility in pregnancy.

### Germline Transgenic Approaches

These approaches used traditional transgenic techniques to manipulate the normal expression of genes thought to be involved in the generation of PE. As with any molecular manipulation that results in overexpression or ablation of genes at the germline level, *both* the placenta and fetus are affected and thus the contribution of the fetal knockout to any pathology or phenotype must be considered (Hemberger et al., 2020).

#### Storkhead Box 1 Overexpression

Storkhead Box 1 (STOX1) is a transcription factor that is structurally and functionally similar to the forkhead box family and has shown to be involved in regulating proliferation and migration in a choriocarcinoma cell culture model for trophoblasts (He et al., 2020). Polymorphisms in STOX1 have been reported as a risk factor for early-onset PE in studies of Dutch and Turkish populations; however, this association was not confirmed in a separate Dutch study, in studies of Finnish or Korean populations, nor in mothers with European-ancestry (van Dijk et al., 2005; Berends et al., 2007; Iglesias-Platas et al., 2007; Kivinen et al., 2007; Kim et al., 2009; Pinarbasi et al., 2020). In addition, levels of STOX1 expression were not associated with PE pathology (Iglesias-Platas et al., 2007; Kivinen et al., 2007).

Wild-type FVB/N female mice crossed with transgenic males overexpressing STOX1 systemically using a cytomegalovirus promoter, develop severe maternal hypertension with parturitional resolution, proteinuria, increased serum angiogenic factors, and kidney alterations (Doridot et al., 2013). Alterations in glycogen trophoblast location in the placenta were found in only one of two transgenic lines developed. A higher transgene copy number was needed to exact this placental phenotype, suggesting that the effects are dependent upon achieving a minimum level of STOX1 overexpression (Doridot et al., 2013). However, no differences were observed in the gross morphology of STOX1 placenta at E16.5. Fetal growth restriction occurred in STOX1 hemizygous mice and litter size was reduced (Doridot et al., 2013; Collinot et al., 2018). While several PE-like symptoms were reported, the systolic blood pressure in STOX1 hemizygous mice became significantly elevated almost immediately after mating occurred and prior to implantation at E3.5; well before the placenta had begun to develop (Figure 1). These results suggest that placenta may not be essential for the some of the pathologies observed in the STOX1 overexpression approach and suggests the possibility that some subtypes of PE may not be necessarily dependent on the placenta as the organ of origin.

#### Indoleamine 2,3-Dioxygenase Knockout

Indoleamine 2,3-dioxygenase (IDO) is a cytosolic, heme-containing protein present in most mammalian tissues and catalyzes the rate-limiting step in tryptophan degradation. IDO is a critical enzyme in T-cell-mediated immune response (Selvan et al., 2016). Decreased IDO mRNA, protein, and activity have been reported in placentas from preeclamptic pregnancies (Santoso et al., 2002; Kudo et al., 2000; Kudo et al., 2003). Santillan et al. (2015) utilized Cre-lox technology and found that pregnant IDO knockout mice have significant renal pathology with glomerular endotheliosis and increased urinary protein, as well as endothelial dysfunction in the aortic arteries. Fetuses from IDO knockout pregnancies were significantly smaller and exhibited fetal growth restriction. Serum and placenta levels of inflammatory cytokines, angiogenic factors, and HIF-1α were not assessed. This approach exhibits some PE-like pathologies; however, no differences in placental morphology were present and pregnant IDO knockout mice did not exhibit elevated blood pressure (Santillan et al., 2015). The IDO knockout approach does not reproduce all of the classical symptoms of PE in humans, however, the model may be useful in examining placental insufficiency and renal dysfunction during pregnancy.

#### ELABELA Knockout

ELABELA (ELA), encoded by the *APELA* gene, is an endogenous peptide ligand for the apelin receptor (APJ) (Xu et al., 2018). ELABELA has an important role in numerous developmental processes including gastrulation, heart morphogenesis, bone formation, skeletal development, and angiogenesis (Xu et al., 2018). ELABELA knockout mice develop elevated BP during pregnancy, beginning at E16 with parturitional resolution, proteinuria, poor placental invasion, and defective placental angiogenesis (Ho et al., 2017). Fetal weight was reduced and placentas exhibited gene expression increases consistent with inflammatory involvement (Ho et al., 2017; Pritchard et al., 2018). ELABELA mice also have increased mRNA levels of placental angiogenic and anti-angiogenic genes at late gestation, but elevated maternal serum levels were not found (Ho et al., 2017). Elevated levels of placental HIF-1α protein were also present in these mice (Ho et al., 2017). Gene ablation of ELABELA in mice produces a phenotype that resembles early-onset PE in humans; however, major fetal cardiovascular malformations occurred in nearly 50% of these fetuses by E10.5 with significantly increased fetal demise.

Studies in human tissue have not found an association of ELABELA with early-onset PE, as placental mRNA and circulating ELABELA levels were not different from normal pregnancies (Pritchard et al., 2018; Zhou et al., 2019). However, results for late-onset PE are conflicting: Zhou et al. (2019) showed a decrease in placental and serum levels of ELABELA in late-onset; whereas Panaitescu et al. (2020) reported increased plasma ELABELA in late-onset. The reasons for this discrepancy and the relevance of ELABELA to late-onset PE remain unclear.

#### Catechol-*O*-Methyltransferase Knockout

Catechol-*O*-methyltransferase (COMT) is an enzyme that catalyzes the *O*-methylation and degradation of catecholamines and other catechol-containing compounds (Zhu, 2002). Genetic polymorphisms in human maternal and fetal COMT have been reported to be associated with decreased placental COMT activity and the development of PE (Hill et al., 2011; Pertegal et al., 2016). COMT knockout mice develop the PE-like features of elevated blood pressure with parturitional resolution and proteinuria (Kanasaki et al., 2008). However, placental development and trophoblast lineages of the labyrinth and junctional zone, as well as, the spongiotrophoblasts and giant cells were normal compared to control; although some vascular lesions and thrombotic deposits were noted (Kanasaki et al., 2008). Litter size and placental efficiency were increased in COMT deficient mice compared to control. Although COMT knockout mice delivered early at E19, fetal growth restriction was not present (Kanasaki et al., 2008). COMT mice also had significantly elevated placental HIF-1α and sFLT-1 protein.

Placenta and serum levels of COMT are not decreased or associated with PE in humans and thus the clinical relevance of the COMT knockout as an approach to simulate PE in mice is unclear (Palmer et al., 2011; Pritchard et al., 2018). However, an association between COMT genotype and blood pressure has been reported in a human study and the expression and activity of COMT has been associated with blood pressure in spontaneously hypertensive rats and Dahl salt-sensitive rats (Tsunoda et al., 2003; Hagen et al., 2007; Hirano et al., 2007). COMT knockout mice may be better suited to study the impact of COMT deficiency in the development of hypertension and hypertension during pregnancy.

#### Complement Component 1q Knockout

Complement component 1q (C1q) is part of the innate immune system and activates the classical complement pathway by recognizing and binding to the antigen-antibody complex. The C1q protein has been shown to be widely distributed in human decidual stroma, is locally synthesized by migrating extravillous trophoblasts, and has an important role in trophoblast invasion and vascular remodeling of the spiral arteries (Agostinis et al., 2010; Chighizola et al., 2020). Serum levels of C1q have been shown to be reduced in maternal serum and plasma of PE pregnancies (Agostinis et al., 2016; Pierik et al., 2020). A different study found that in early-onset cases, C1q protein levels in the placenta were significantly lower than in late-onset PE (Lokki et al., 2014; Pierik et al., 2020).

Knockout of C1q in mice results in a significant elevation in blood pressure on E13 during pregnancy and increased albumin:creatinine ratio at E9 (Figure 1) (Singh et al., 2011). Placental structure was affected and showed impaired labyrinth development, reduced decidual vessel remodeling, decreased trophoblast invasion, and significantly reduced placental blood flow (Singh et al., 2011). Knockout animals also had decreased placental VEGF and elevated levels of sFLT-1 (Singh et al., 2011). The protein levels of HIF-1α were not analyzed. Increased fetal resorption, reduced fetal weight, and decreased litter size were noted (Singh et al., 2011; Agostinis et al., 2017). Interestingly, when wild-type females were crossed with C1q-deficient males, increased fetal resorption, and decreased fetal weight were recapitulated, similar to the homozygous C1q-deficient crossings, while C1q-deficient females crossed with wild-type males did not recapitulate the phenotype. These data indicate that maternal C1q is not required for placental development or embryonic survival and suggests that defective fetal production of C1q may be involved in the pathogenesis of PE. However, the very early renal dysfunction (E9) prior to hypertension (E13) observed in C1q-deficient mice suggests the possibility of PE subtypes with kidney involvement prior to hypertension.

### Exogenous Agent Approaches

The approaches outlined in this section all involve the administration of an exogenous agent to pregnant mice to induce PE-like pathologies. The large majority of these agents modulate the immune system and are typically introduced after placental development has occurred, suggesting that these approaches would be more representative of late-onset PE.

#### AT1-AA

Autoantibodies against the angiotensin II type 1 receptor (AT1-AA) are elevated in some women with PE and have been associated with other pathologies like systemic sclerosis, tissue fibrosis, hypertension, and renovascular disease, among others (Campbell et al., 2018). These autoantibodies activate the angiotensin II type 1 receptor, transduce signals via the MAPK/ERK pathway, and result in increased vasoconstriction and elevated blood pressure (Campbell et al., 2018; Marshall et al., 2018).

Zhou et al. (2008) reported significantly increased blood pressure after pregnant mice were administered angiotensin II type 1 receptor autoantibodies isolated from preeclamptic women. Proteinuria and glomerular swelling were present when analyzed at E18.5. Placentas were significantly smaller than normal and fetal growth restriction was noted. Extensive cellular disorganization of the labyrinth zone was present following administration of isolated IgG from PE women to pregnant mice. The circulating levels of sFLT-1 and sENG were significantly increased in maternal serum on E18 after treatment with PE-IgG on E13 and E14 (Zhou et al., 2008). Parturitional resolution of the PE-like phenotype was not reported.

Angiotensin II receptor inhibitors are contraindicated in pregnancy due to their teratogenic effects and other interventions, like plasmapheresis, have been ineffective in treating PE (McCarthy et al., 2011). Some features of this approach are not pregnancy specific, as administration of IgG from PE women to non-pregnant mice significantly increased blood pressure on a time course similar to pregnant animals, with increased blood pressure occurring 4-5 days after treatment (Zhou et al., 2008). Additionally, non-pregnant animals did not show increased sFLT-1 levels, renal damage, or proteinuria. These data suggest that if autoantibodies to the angiotensin type II receptor are present in the absence of pregnancy then the women would have high blood pressure chronically as a result of the autoimmune condition and that delivery of the baby would not resolve the symptoms.

#### Lipopolysaccharide

Lipopolysaccharides (LPS) are present on the outer membrane of Gram-negative bacteria and binds toll-like receptor 4 complex in numerous cell types. In immune cells, LPS can trigger the release of proinflammatory cytokines and the generation of reactive oxygen species (Mittal et al., 2014). LPS are the prototypical endotoxin and can induce systemic inflammation. LPS administration has been used in PE research to stimulate an inflammatory state that may be similar to what occurs in PE.

Li et al. (2018) injected LPS in CD1 (ICR) mice daily, from E7.5 to E17.5, and observed significantly elevated blood pressure from E8.5-E18.5 (Figure 1). At E18.5, fetal blood vessel area in the placenta and trophoblast invasion and spiral artery remodeling were reduced. Kidney damage with fibrin deposits and swollen glomeruli were observed, as was proteinuria. Fetal and placental weights were significantly reduced; however, the impact on litter size was not reported. As would be expected, LPS treatment resulted in elevated levels of proinflammatory cytokines (IL-6, IL-1β, and TNF-α), as well as sFLT-1 in the placenta and serum (Li et al., 2018).

In a different study, Huai et al. (2018) administered LPS as a single, ultra-low dose at E7 in C57BL/6J mice. Maternal blood pressure was significantly elevated 1 day after injection and remained elevated throughout pregnancy (Huai et al., 2018). Urinary protein was elevated, but albumin-to-creatinine ratio was not reported. Fetal growth and placental efficiency were significantly reduced with this approach, compared to control but litter size remained unchanged (Huai et al., 2018). Pathological changes were noted in the maternal liver and kidney, as well as the placenta (Huai et al., 2018).

Administration of LPS leads to some pathological features associated with early-onset PE or late-onset depending on when it is given, with hypertension occurring immediately following administration (Zhou et al., 2017; Huai et al., 2018; Li et al., 2018). Notably, the hypertension elicited in pregnant mice, when given LPS early, occurred before completion of placentation (Figure 1). Lipopolysaccharide induces systemic inflammation in pregnant and non-pregnant animals suggesting that the phenotype created in the low-dose LPS approach is likely not pregnancy specific and more closely recapitulates an increased chronic inflammatory state than mechanisms underpinning the initiation PE (Faas et al., 1994; Beller, 1995; Huai et al., 2018; Li et al., 2018).

#### Tumor Necrosis Factor Alpha

Tumor necrosis factor alpha is the prototypical, pro-inflammatory cytokine of the tumor necrosis superfamily. This multifunctional signaling molecule is involved in the regulation of a wide variety of biological processes including cell proliferation, differentiation, apoptosis, and inflammation (Kalliolias and Ivashkiv, 2016). Increased TNF-α levels have been found in PE maternal circulation compared to normal pregnancies and non-preeclamptic, hypertensive pregnancies (Hayashi et al., 2005; Cackovic et al., 2008; Founds et al., 2008; Tosun et al., 2010). The serum levels of TNF-α were significantly increased in early-onset PE, compared to late-onset PE (Peraçoli et al., 2013).

Continuous daily infusion of TNF-α in mice using an osmotic pump and beginning on E13.5, led to elevated blood pressure on E17 and E18 (Bobek et al., 2015). Total urinary protein was increased; however, the albumin-to-creatinine ratio, a more direct measure, was not determined. Fetal weight and litter size at E17 were normal compared to saline control (Bobek et al., 2013, 2015). Placental HIF-1α mRNA and protein at E17 were elevated in the labyrinth and junctional zone; however, there was no induction of serum or placental antiangiogenic sFLT-1 by TNF-α (Bobek et al., 2015). Neither placental lineages nor parturitional resolution were analyzed (Bobek et al., 2015). However, a subsequent study investigating placental macrostructure following TNF-α treatment using magnetic resonance microscopy did not find alterations in the labyrinth, junctional zone, or decidual volumes compared to control (Bobek et al., 2018).

As pregnancy is thought to lead to increased inflammation with circulating proinflammatory cytokine levels increasing throughout gestation, the involvement of TNF-α in PE is probable. Continuous administration of TNF-α induces some pathological features of PE in pregnant mice probably due to exacerbation of the already pro-inflammatory state and suggests a role for inflammatory cytokines in the generation of the maternal syndrome. However, TNF-α and other proinflammatory cytokines as a central mediator of PE remains inconclusive and are more likely secondary downstream effectors of initiating events.

#### LIGHT

LIGHT (TNFSF14) is a proinflammatory cytokine and a member of the TNF superfamily. LIGHT exists as two forms: a plasma membrane bound protein and a soluble form that is secreted from cells (Granger et al., 2001). In both humans and mice, LIGHT is an immune signaling molecule that activates two specific cellular receptors, lymphotoxin β-receptor (LTβR) and herpes virus entry mediator (HVEM) (Zhai et al., 1998). The LIGHT protein, as well as both of its receptors, are present on trophoblast and endothelial cells of the placenta (Phillips et al., 2001; Gill et al., 2002, 2007). Significantly higher levels of LIGHT protein have been found in the maternal serum of preeclamptic patients, particularly in the third trimester (Wang et al., 2014; Hirashima et al., 2018). Also, a soluble LIGHT decoy receptor, DcR3 is present in human placental tissue and serum levels of DcR3 are significantly decreased in the third trimester of preeclamptic pregnancies (Lin and Hsieh, 2011; Hsieh and Lin, 2017; Yeh et al., 2019).

Administration of soluble LIGHT to pregnant C57Bl/6 mice at E13.5 and E14.5, after normal placental development has occurred, led to a significant increase in blood pressure by E17.5 and proteinuria with renal abnormalities (Wang et al., 2014). Blood pressure and urinary protein were substantially reduced postpartum and were associated with decreased levels of LIGHT in circulation. Placental calcifications and increased apoptosis were noted in the labyrinth; however, trophoblast lineage analysis was not reported (Wang et al., 2014). LIGHT also induced smaller placentas and fetal growth restriction at E18.5. Serum and placental sFLT-1, as well as placental HIF-1α mRNA, were significantly increased in these mice, although protein levels were not reported (Wang et al., 2014; Iriyama et al., 2015).

Injection of LIGHT in non-pregnant C57Bl/6 mice induced high blood pressure but not proteinuria, indicating that at least some of the PE-like features in this approach are not pregnancy specific (Wang et al., 2014; Luo et al., 2016). Additionally, mice do not express DcR3; however, the lack of decoy receptor may approximate the human condition, where levels of DcR3 are significantly reduced in third trimester of human PE (Lin and Hsieh, 2011; Wang et al., 2014; Hirashima et al., 2018; Yeh et al., 2019). The LIGHT approach may be useful in studying the effect of maternal inflammation in pregnancy.

#### L-NAME

The last member of the exogenous agent group is L-NAME (nitro-L-Arg-methyl ester), a pan-nitric oxide (NO) synthase (NOS) inhibitor. NO is a potent vasodilator that is synthesized from the amino acid, L-arginine, by NOS, and has been hypothesized to be involved in generation of PE due to its role in controlling vascular tone. Yoshikawa et al. (2017) tested the inhibition of NO production in pregnancy and its involvement in the generation of PE like symptoms. L-Name administered to pregnant C57Bl/6 mice from E11 throughout pregnancy resulted in significantly elevated blood pressure as early as E13 (Figure 1) (Yoshikawa et al., 2017). The increased high blood pressure was maintained at E16 and was accompanied with proteinuria and glomerulosclerosis. Placental and fetal weight were significantly reduced at E17.5 and litter sizes were normal. Alterations in the decidua, junctional zone or labyrinth were not present in mice treated with L-NAME; however, the maternal and fetal blood spaces were narrowed. Serum and placenta levels of PIGF or sFLT-1 were not different. Expression and protein levels of HIF-1α or proinflammatory cytokines were not analyzed nor was parturitional resolution of the maternal syndrome.

Administration of L-NAME to non-pregnant mice induced significant blood pressure elevation with aortic vascular contractions, indicating the phenotype generated in pregnant mice is not pregnancy specific (Yadav et al., 2019). Additionally, the relevance of the L-NAME approach to the human condition is unclear as “defects in NO production have not been consistently demonstrated in PE” (McCarthy et al., 2011) and the involvement of NOS in PE is uncertain (Samangaya et al., 2009; McCarthy et al., 2011; Erlandsson et al., 2016; Cushen and Goulopoulou, 2017; Hitzerd et al., 2019; Gatford et al., 2020). While chronic inhibition of NO production can some produce clinical manifestations of PE, this approach does not appear to be “a physiologically accurate representation of the pathophysiology of PE” (Marshall et al., 2018).

### Surgical Approach

#### Reduced Uteroplacental Perfusion Pressure

The reduced uteroplacental perfusion pressure (RUPP) approach utilizes physical restriction of blood flow to the placenta and fetus by clamping or ligating the maternal arteries during pregnancy. Near complete occlusion of these arteries significantly decreases blood flow to the fetus, resulting in placental ischemia and a dramatic reduction in systemic maternal blood flow. Intapad et al. (2014) performed RUPP in C57Bl/6J mice by clamping the ovarian and uterine arteries, as well as the abdominal aorta, at E13. This approach resulted in the development of hypertension with fetal growth restriction, as well as increased serum, but not placental, sFLT-1 at E18. No difference in proinflammatory TNF-α or angiogenic VEGFα levels were noted and histological examination of the placenta and trophoblast lineages was not performed. In addition, proteinuria was not present, perhaps because C57Bl/6 mouse strain is more resistant to renal injury than others, such as ICR (Ishola et al., 2006; Intapad et al., 2014; Rabe and Schaefer, 2016).

A different group, Fushima et al. (2016), bilaterally ligated the ovarian and uterine arteries in ICR mice on E14.5, which resulted in decreased placental weight, fetal growth restriction, and a substantial increase in fetal demise (Sekimoto et al., 2020). These mice had elevated blood pressure as early as 1 day after surgery, increased urinary albumin with proteinuria, and significant endotheliosis at E18.5 (Fushima et al., 2016). Maternal serum sFLT-1, VEGF, and PIGF protein levels were similar to controls; however, placental sFLT-1 and HIF-1α mRNA were significantly elevated in RUPP mice (Sekimoto et al., 2020). While expression of pro-inflammatory cytokine, IL-6, and platelet levels were unchanged, an elevation of liver enzymes in the serum was reported (Fushima et al., 2016). As the ligation occurred at E14.5, placental development would be expected to have been normal, and as such trophoblast lineages were not assessed in these studies. Notably, positional effects of ligation were seen, with more fetal resorption and lower fetal weights observed the closer the fetus was to the site of ligation, indicating that the technique can produce variable fetal effects, potentially confounding interpretation (Fushima et al., 2016).

Analysis of placental development and trophoblast lineages was reported by Natale et al. (2018) after inducing RUPP via bilateral ligation of the uterine arteries at E14.5 in ICR mice. Blood pressure and proteinuria were not reported in this study (Natale et al., 2018). Fetal growth restriction at E18.5 was present and was accompanied by increased placental weight and decreased placental efficiency. Fetal resorption and litter size did not change (Natale et al., 2018). The level of VEGFα in the whole placenta was unchanged; however, the level of VEGFα in the labyrinth was reduced. Increased placental HIF-1α mRNA was also observed; however, proinflammatory cytokines, TNF-α and IL-6 were unchanged (Natale et al., 2018). Interestingly, an elevated number of trophoblast giant cells and a reduced junctional zone with fewer spongiotrophoblast and glycogen cells were noted at E18.5 (Natale et al., 2018). Furthermore, a reduced pericyte presence was noted in the labyrinth of the RUPP placenta. These mice had normal maternal blood spaces but did have larger fetal blood spaces in the labyrinth at E18.5. Natale et al. have shown that placental alterations can occur after normal development, thus demonstrating the necessity to assess trophoblast lineages in other “late-onset” approaches.

The RUPP approach produces some pathological effects similar to late-onset PE with fetal growth restriction. As RUPP surgery is performed after placental development has occurred, spiral artery remodeling should have occurred normally and would not be useful for the study of early alterations in the immune system, trophoblast invasion, endothelial function, or placentation, but would be best used for studies of placental ischemia and placental insufficiency.

### Viral Approaches

Several reported methods have utilized viral induction of target genes to generate PE-like symptoms in mice. Two different target genes, sFLT-1 and HIF-1α, have been used with two different viral approaches: a systemic adenoviral infection approach and a trophoblast-specific lentiviral approach. The target genes and the viral approaches generate different pathologies in mice. In the *systemic adenoviral approach*, adenovirus was administered via tail vein injection in early pregnancy, around E7-8 and resulted in systemic, maternal exposure to adenovirus with the major sites of infection including the liver, lung, and spleen. Adenoviral uptake and misexpression of target genes in the liver can result in maternal, pseudo-viral hepatitis, and raises questions about the relevance of the systemic adenoviral approach to the more typical forms of PE in humans (Venditti et al., 2014).

The *trophoblast-specific lentiviral transgenesis approach* involves the infection of isolated blastocysts to express the target gene only in the trophoblast portion of the placenta. *This sophisticated approach obviates the systemic viral exposure and targets transgene expression exclusively to placental trophoblasts without affecting the mother and fetus* (Georgiades et al., 2007; Malashicheva et al., 2007; Okada et al., 2007; Lee et al., 2009; Morioka et al., 2009; Fan et al., 2011, 2012; Kumasawa et al., 2011; Kashif et al., 2012; Kaufman et al., 2014; Latos et al., 2015; Chakraborty et al., 2016; Mishima et al., 2016; Muto et al., 2016; Albers et al., 2019). This approach is more physiologically relevant because the placenta is the only organ with genetic manipulation and the initiating event for the induced pathologies. In addition, the lentiviral transgenesis approach prevents the unavoidable off-target or secondary effects from approaches that involve systemic viral infection, administration of exogenous agents, or surgical intervention.

#### Soluble fms-Like Tyrosine Kinase 1

Soluble fms-like tyrosine kinase 1 is a non-membrane bound, splice variant of the VEGF receptor. The sFLT-1 protein lacks the transmembrane domain and the tyrosine kinase domain that allows for VEGF angiogenic signaling. Soluble FLT-1 binds to VEGF and placental growth factor (PIGF, a truncated VEGF homolog secreted by the placenta) in circulation, reducing the amount available and thus, decreasing angiogenic signaling. The levels of sFLT-1 increase throughout normal pregnancy but increase faster and to a higher level in pregnancies complicated by PE (Levine et al., 2004). Placental sFLT-1 mRNA and protein were found to be increased in preeclamptic pregnancies and were associated with decreased levels of circulating VEGF and PIGF (Maynard et al., 2003).

Administration of adenovirus expressing sFLT-1 in pregnant CD-1 (ICR) mice on E8 of gestation resulted in increased plasma sFLT-1, increased mean blood pressure, and decreased fetal and placental weight on E18 (Lu et al., 2007). Litter sizes were unchanged and placental abnormalities were not investigated; however, protein deposits in the collecting tubules were observed, suggesting renal damage. This study used a truncated form of sFLT-1 that lacks some N-terminal domains and is less homologous to the predominant form of sFLT-1 in humans (Suzuki et al., 2009). More recent studies used the full length sFLT-1 protein in pregnant mice and found that PE-like symptoms, specifically hypertension, proteinuria, and decreased circulating VEGFA occurred with high doses of virus (Suzuki et al., 2009; Bergmann et al., 2010). Higher doses of full length sFLT-1 adenovirus also resulted in severe renal endotheliosis (Bergmann et al., 2010). Lower doses of adenovirus did not result in a phenotype different control (Suzuki et al., 2009).

Systemic, adenoviral mediated sFLT-1 expression during murine pregnancy reproduces features similar to the human condition – indicating that antiangiogenic signaling might be involved in PE etiology. However, the elevation of liver enzymes and hemolysis due to the involvement of the maternal liver, serving as the primary site of adenoviral infection, suggests that this approach more closely resembles the rare PE subtype of HELLP (hemolysis, elevated liver enzyme, and low platelet) syndrome or pseudo-viral hepatitis than typical PE (Bergmann et al., 2010; Szalai et al., 2015; Oe et al., 2018). In addition, reports using systemic intravenous adenoviral infection in cancer studies have demonstrated that this approach can be hepatotoxic (Huard et al., 1995; Mahasreshti et al., 2001).

To better model the human condition and establish the placenta as the major pathological determinant, Kumasawa et al. (2011) used a different approach and lentivirally transduced the trophectoderm of isolated murine blastocysts to specifically express human sFLT-1 (hsFLT-1) in the trophoblasts of the placenta. Trophoblast-specific overexpression of hsFLT-1 in mice resulted maternal hypertension beginning at E16.5 with proteinuria at E18.5 and parturitional resolution (Kumasawa et al., 2011). The placental-specific expression of hsFLT-1 mice exhibited renal glomerular sclerosis, in conjunction with the proteinuria at E18.5 (Kumasawa et al., 2011). Fetal development and placental structure were also perturbed in hsFLT-1 pregnant mice. Fetal growth restriction was present compared to EGFP controls when evaluated at E18.5. Placental weight was also reduced in hsFLT-1 overexpression and was associated with suppressed vascular bed development and reduced maternal and fetal blood spaces at E13.5.

Besides the hsFLT-1 splice variant used in Kumasawa et al., other variants have been shown to have relevance to PE including other splice variants and protease-mediated cleavage of membrane bound isoforms (Aubuchon et al., 2011). While the trophoblast-specific hsFLT-1 approach leads to expression throughout the fetal part of the placenta, endogenous expression of mouse sFLT-1 in the placenta is restricted exclusively to the spongiotrophoblast cells and this misexpression of hsFLT-1 may have unknown effects that could be involved in the generation of the phenotype (Hirashima et al., 2003).

#### Hypoxia-Inducible Factor 1 Alpha

In both humans and mice, the oxygen levels experienced by the developing fetus and placenta change throughout pregnancy – with a very low oxygen environment characterizing early stages followed by a dramatic increase in oxygen levels just prior to completion of placentation (Caniggia et al., 2000; Gultice et al., 2009; Pringle et al., 2010; Soares et al., 2017; Albers et al., 2019). Proper placental and fetal development are dependent on the oxygen level of their surrounding environment and changes in oxygen level affect many cellular processes, such as differentiation, metabolism, and invasion (Ducsay et al., 2018). Developmentally abnormal oxygen levels or the disrupted ability of trophoblast cells to sense and respond to it may have detrimental effects on the fetus and maintenance of pregnancy. The cellular response to changes in environmental oxygen is predominately mediated by the transcription factor, HIF-1α. HIF-1α is highly dynamic, as its mRNA is constitutively synthesized, but the protein is regulated by oxygen-dependent degradation. When trophoblast cells experience maternal arterial oxygen levels (∼12% O_2_) at the end of the first trimester in humans or E10.5 in mice, the HIF-1α protein is rapidly degraded (about 5 min), and prevents its continued activity (Berra et al., 2001; Caniggia and Winter, 2002; Rajakumar et al., 2004; Ietta et al., 2006; Pringle et al., 2007, 2010; Kaelin and Ratcliffe, 2008; Gultice et al., 2009; Semenza, 2012; Zhao et al., 2020).

Hypoxia-inducible factor 1 alpha is essential for proper embryonic development, as knockout studies in mice are lethal by E10.5 (Iyer et al., 1998). Numerous other studies have demonstrated that HIF-1α is a critical regulator of many aspects of placental development, including cell fate, trophoblast differentiation and invasion, lineage determination, and vascular remodeling (Gultice et al., 2009; Kanasaki and Kalluri, 2009; Rolfo et al., 2010; Roberts and Escudero, 2012; Redman et al., 2014; Gathiram and Moodley, 2016; Albers et al., 2018). Knockout of VHL, a protein necessary for normal HIF-1α degradation, is also lethal between E10.5-E12.5 due to impaired placental vasculogenesis (Gnarra et al., 1997). Also, placental knockdown of another protein required for normal HIF-1α degradation, EGLN1 (PHD2), resulted in smaller fetal weights at E14.5 and disruption of placental structure with more connective tissue, extension of spongiotrophoblast cells, and an increased number of necrotic cells in the labyrinth (Ozolins et al., 2009). *In humans, elevated levels of placental HIF-1*α, *beyond the first trimester, are strongly associated with preeclampsia* (Caniggia and Winter, 2002; Soleymanlou et al., 2005; Zamudio et al., 2007; Rolfo et al., 2010; Tal, 2012; Iriyama et al., 2015; Korkes et al., 2017; Park et al., 2018; Rana et al., 2019). The observed elevation of placental HIF-1α in these pregnancies associated with PE is specific to the trophoblasts and suggest that a precise balance in HIF-1α signaling is required for appropriate placental development and that deviation from the normal physiological window could be pathological and may be a central factor in PE etiology.

An initial investigation into prolonged HIF-1α and the development of PE-like symptoms was performed by Tal et al. (2010) using systemic adenoviral infection for HIF-1α overexpression in pregnant mice. Pregnant C57Bl/6J mice were injected with adenoviral HIF-1α at E8 and were evaluated on E18. HIF-1α pregnant dams exhibited elevated blood pressure, proteinuria, and glomerular endotheliosis (Tal et al., 2010). Placental and fetal weights were reduced, suggesting fetal growth restriction, but litter size was unchanged. Placental histology showed dilation of the maternal and fetal blood vessels and increased volume of maternal and fetal blood spaces. Numerous calcifications, fibrin thrombi, and infarction were also noted. Morphometric analysis indicated no disruptions in the decidua, junctional zone, in the labyrinth, and no changes in trophoblast invasion or spiral artery remodeling (Tal et al., 2010). Serum sFLT-1 and sENG were significantly increased in pregnant HIF-1α animals on E18. The serum levels of sFLT-1 and sENG were also transiently increased in non-pregnant animals 4-8 days after adenoviral infection.

Tal et al. (2010) concluded that the systemic adenoviral HIF-1α approach approximates the HELLP syndrome seen in humans, as the major sites of adenoviral HIF-1α infection were the liver, spleen, and lung. Adenovirus administration coincided with decreased hematocrit and platelet levels, increased serum liver enzymes, and increased serum lactate dehydrogenase levels in both pregnant and non-pregnant mice (Tal et al., 2010). Similar to the adenoviral sFLT-1 approach, some of the symptoms generated in the systemic adenoviral HIF-1α approach also occur in non-pregnant animals, because the major biological determinant in the adenoviral approach is not the placenta. Thus, this approach most likely represents a model of pseudoviral hepatitis during pregnancy or HELLP, rather than the more typical forms of PE (Huard et al., 1995; Mahasreshti et al., 2001; Tal et al., 2010; Oe et al., 2018).

**FIGURE 3:**
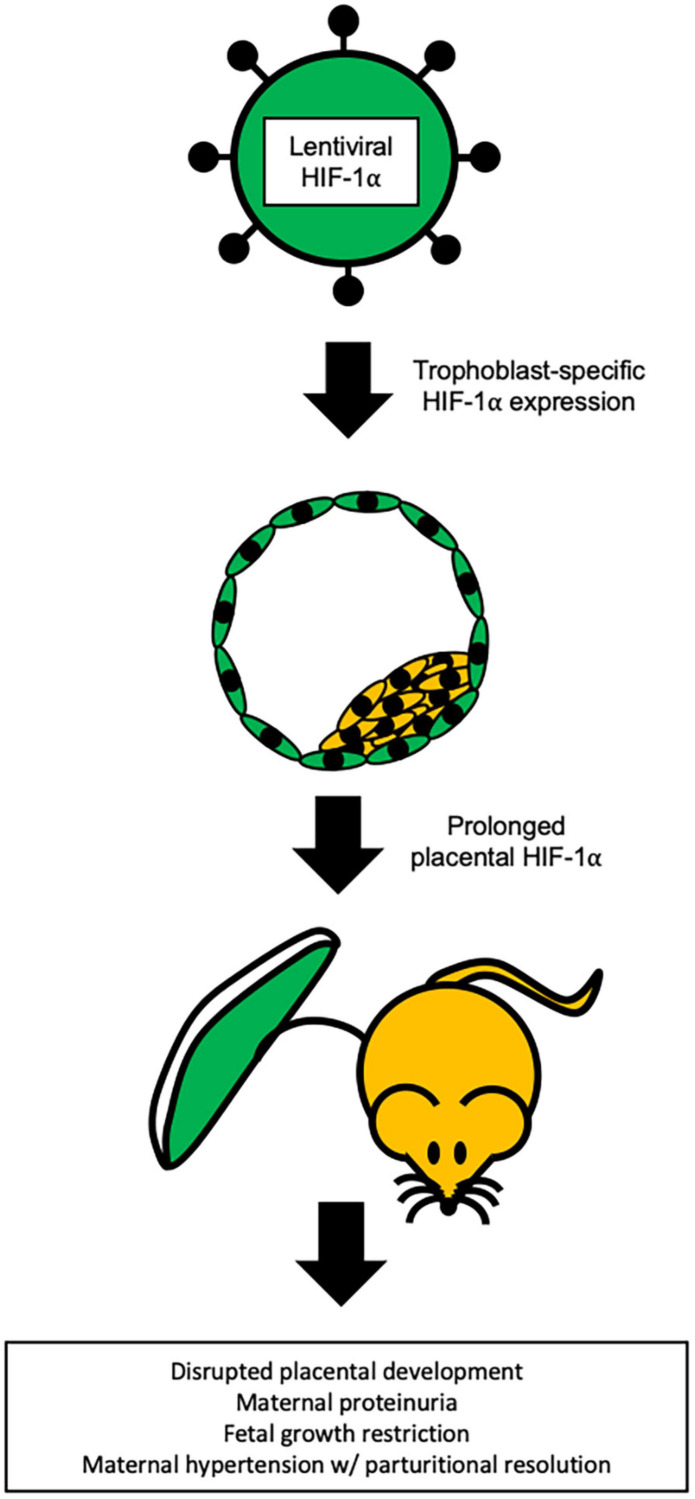
Trophoblast specific HIF-1α mouse model of PE. Representation of the lentiviral-mediated trophoblast-specific HIF-1α mouse model of preeclampsia. Prolonged HIF-1α is restricted to the trophoblasts of the placenta and results in pregnancy specific hypertension with parturitional resolution, proteinuria, and fetal growth restriction (Kaufman et al., 2014; Albers et al., 2019).

To avoid the confounding variables of the adenoviral approach and to the better understand the impact of HIF-1α in placental development and pregnancy, Albers et al. (2019) utilized lentiviral-mediated, trophoblast-specific transgenesis to prolong placental HIF-1α beyond its normal developmental window (Figure 3). This approach resulted in significantly increased systolic blood pressure with parturitional resolution, proteinuria, and glomerular endotheliosis – the classic hallmarks of PE (Albers et al., 2019). At birth, HIF-1α offspring exhibited fetal growth restriction, but no changes in litter size nor placenta weight were observed. In addition, significant physiological alterations in placental differentiation were noted and included reduced branching morphogenesis, alterations in maternal and fetal blood spaces, and failure to remodel the maternal spiral arteries. Placental and serum levels of inflammatory cytokines were not evaluated, and serum sFLT-1 was not increased compared to controls (Albers et al., 2019). However, levels of sFLT-1 in the placenta have been shown to be highly variable across litters in mice, with a more than two-fold difference in expression between litters of normal pregnancies and may contribute to the varying reports of sFLT-1 level changes in mice (Surmon et al., 2014a). While in humans, the levels of placental sFLT-1 mRNA showed variability with location of tissue extraction in both normotensive and preeclamptic pregnancies (Surmon et al., 2014b).

The trophoblast-specific HIF-1α approach uses a gene that is highly associated with the human condition and when prolonged outside of its normal developmental window, produces an array of pathologies in mice that strikingly resemble the hallmarks of early-onset PE in humans (Albers et al., 2019). Moreover, HIF-1α is not misexpressed because this lentiviral transgenesis approach targets only the trophoblasts and not fetus, decidua, or maternal organs. This mimics the endogenous HIF-1α expression pattern where HIF-1α is present throughout the placental trophoblasts (Pringle et al., 2007, 2010; Zhao et al., 2020). Use of trophoblast-specific HIF-1α gene transfer is a significant technological advance in the generation of a model that closely recapitulates early-onset PE in humans.

## Conclusion

Many approaches have been put forth over the years as potential mouse models for PE. These models have advanced the understanding of how physiological abnormalities identified in human PE are involved in the generation of the condition. Some of the older approaches are not as widely used now because new information has changed our understanding of PE and thus altered what is considered important and relevant. The advent of new technologies and techniques has also changed how researchers create new models and has drastically improved the simulation of PE in mice (Georgiades et al., 2007; Malashicheva et al., 2007; Okada et al., 2007; Lee et al., 2009; Morioka et al., 2009; Fan et al., 2011, 2012; Kumasawa et al., 2011; Kashif et al., 2012; Kaufman et al., 2014; Latos et al., 2015; Chakraborty et al., 2016; Mishima et al., 2016; Muto et al., 2016; Albers et al., 2019).

Preeclampsia is heterogenous in its presentation and a single approach will likely be insufficient to recapitulate the variety of phenotypes present in women and necessitates continued model development. As the existence of at least two PE subtypes based on gestational time is well-established, it is reasonable to consider the possibility that PE may become more stratified as different subtypes, like molecular subgroupings, could be identified in the future and it will be necessary to determine how these new subtypes relate to the current and future animal models of PE.

The physiological features assessed for pathology in mouse models of PE vary greatly between reports, which can hinder interpretation because the impact of the approach on mother and fetus may not be fully captured. Meaning that some pathologies that may be present are still unknown because they were not assessed. Therefore, a standardization of the pathological features to be evaluated when using animal models of PE should be established with the goal of achieving parity between investigations and providing clarity when evaluating models. A standardized set of physiological features will also establish guidelines for determining improvement in therapeutic investigations. *Thus, we propose the phenotypic criteria listed in Table 2 as a starting point for discussions on establishing a standardization*. The proposed features to be evaluated in mouse models of PE are based on physiological alterations associated with PE in women (Table 2). The goal of this work has been to describe and discuss the current state of PE mouse models. Hopefully, standardizing the phenotypic features to be evaluated in PE animal models will improve experimental design, refine results, and clarify interpretation to provide a more common foundation for future preclinical and translational studies.

**TABLE 2:** Proposed standardization and reported phenotype in mouse models of PE.

**Phenotypic features for PE mouse models**	**DBA/2 × CBA/J**	**STOX1 KO**	**IDO KO**	**ELABELA KO**	**COMT KO**	**C1q KO**	**AT1-AA**	**LPS**	**TNF-α**	**LIGHT**	**L-NAME**	**RUPP**	**Adv sFLT-1**	**Lenti sFLT-1**	**Adv HIF-1α**	**Lenti HIF-1α**
Pregnancy-specific maternal hypertension		X	–	X	X	X								X		X
Parturitional resolution of maternal hypertension		X		X	X	X				X				X		X
Placental lineage analysis	X	X/–	–		–	X	X	X	–		–	X		X	–	X
Deficits in maternal spiral artery remodeling				X	–	X		X								X
Alterations in maternal/fetal placental blood spaces	X			X	–	X		X			X	X		X	X	X
Angiogenic alterations in placenta or maternal serum (sFLT-1, VEGF, PIGF, sENG)	X	X		X/–	X	X	X	X	–	X	–	X/–	X	X	X	–
Elevated/prolonged placental HIF-1α				X	X				X	X		X			X	X
Altered inflammatory cytokines in placenta or maternal serum (IL1-beta, IL-6, TNF-alpha)	X	X		X/–				X				–				
Maternal proteinuria/kidney damage (ACR)	X	X	X	X	X	X	X	X	X	X	X	X/–	X	X	X	X
Maternal liver involvement/damage (AST, ALT)								X				X	X		X	
Fetal weight (FGR)	X	X	X	X	–	X	X	X	–	X	X	X	X	X	X	X
Placental weight							X	X	–	X	X	X	X	X	X	X
Fetal/placental efficiency					X							X				X

*List of proposed pathologies to be evaluated when creating or using a mouse model of preeclampsia and their presence in current models. The list was generated from features associated with preeclampsia in humans. Adv = adenoviral, Lenti = lentiviral, X = reported pathology, – = reported normal, blank indicates that the phenotypic feature was not reported.*

## Author Contributions

All authors listed have made a substantial, direct and intellectual contribution to the work, and approved it for publication.

## Conflict of Interest

The authors declare that the research was conducted in the absence of any commercial or financial relationships that could be construed as a potential conflict of interest.
